# Measles Outbreak among Adults, Northeastern China, 2014

**DOI:** 10.3201/eid2201.151293

**Published:** 2016-01

**Authors:** Ming-Xiang Zhang, Jing-Wen Ai, You Li, Bing-Yan Zhang, Wen-Hong Zhang

**Affiliations:** The Sixth People’s Hospital of Sheyang, Shenyang, China (M.-X. Zhang, Y. Li);; Fudan University, Shanghai, China (J.-W. Ai, B.-Y. Zhang, W.-H. Zhang)

**Keywords:** Measles, viruses, China, adults, disease outbreaks, vaccines

**To the Editor:** In 2005, the World Health Organization (WHO) proposed to eliminate measles in the Western Pacific Region by 2012, and in 2006, China began a 6-year measles elimination campaign. The strategy included a routine 2-dose measles-containing vaccine (MCV) for children 8 months and 18–23 months of age, supplemented by nationwide vaccination activities in 2010 for children born during 1996–2010 (*1*). As a result, China’s measles incidence rate has dropped sharply since 2008 and reached its lowest level (0.46 cases/100,000 population) in 2012 (*2*). However, the rate has risen again since 2012; in 2014, incidence was 3.88 cases/100,000 population (*3*). Shenyang, a hub city in northeastern China, experienced a massive measles outbreak in 2014, and we analyzed the causes and characteristics of this outbreak.

Shenyang Center of Disease Control reported 2,058 confirmed measles cases (1,447 laboratory diagnosed, 611 clinically diagnosed) in 2014 (25.02 cases/100,000 population), much higher than that reported in Shenyang in 2013 (2.33/100,000). Most cases occurred in children 0–1 years of age (487 cases; 1,145.77/100,000), followed by persons 25–30 (227 cases; 28.57/100,000), 30–35 (203 cases; 32.42/100,000), and 35–40 (203 cases; 35.02/100,000) years of age. Among all 2,058 confirmed cases, 438 patients were hospitalized because of measles complications; no deaths were reported.

Within Shenyang, Kangping district had the highest confirmed measles incidence rate (80.59 cases/100,000 persons), followed by Tiexi (38.08/100,000) and Faku (32.2/100,000) districts. The remaining confirmed cases occurred in other districts.

Of the 1,207 adults with confirmed measles, migrant workers (640 cases) and farmers (234 cases) accounted for 72.4% of total cases. All confirmed measles-infected adults were surveyed by questionnaire; 93.0% did not recall receiving MCV or had no history of MCV. All 44 measles virus samples genotyped were genotype H1a.

The most notable characteristic of this outbreak was that adults accounted for more than half of reported cases (Figure). Shenyang conducted citywide supplementary vaccination activities in 2009 directed toward children born during 1995–2009, and among these cohorts (now 5–19 years of age), the incidence rate was lower in this outbreak, proving the efficiency of the supplementary vaccination activities. However, for patients >20 years of age, who were not included in the supplementary vaccination activities, the efficacy of their previous 2-dose vaccines also should have offered protection. Thus, other potential risk factors must exist.

**Figure:**
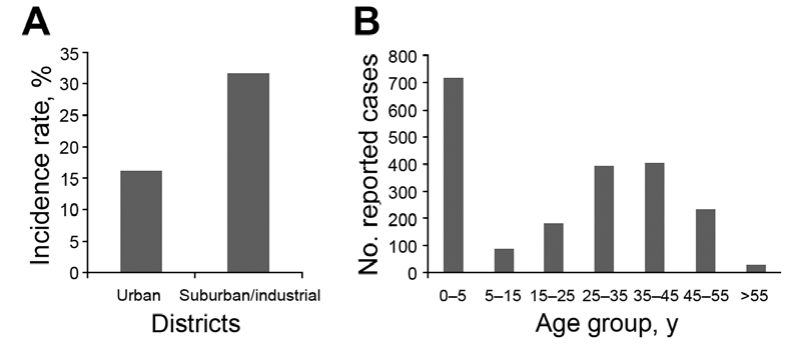
Geographic (A) and age (B) distributions of measles patients, Shenyang, China, 2014.

One risk factor is the limited vaccine coverage. China started the measles vaccine plan in the 1960s, but from 1960 until the 1980s, local vaccination coverage was poor for suburban populations. In recent years, the national reported coverage of both 1-dose and 2-dose MCV have increased from <85% to >98.5% for 2-year-olds (*2*,*4*). However, a door-to-door measles questionnaire survey during an outbreak in Henan province in 2013 reported vaccine coverage of only 80%–90% (*5*). The reason may be that, currently, China calculates vaccine coverage using the number of vaccinated children as the numerator and the number of clinic-registered children as the denominator. This method excludes those who did not register at a community clinic (e.g., because the family breached the 1-child policy and therefore refused registration or because of lack of medical insurance) and thus resulted in higher reported coverage rates. Unvaccinated persons who missed supplementary vaccination activities also possibly became susceptible to measles.

The second characteristic was the higher incidence rates in the suburban than urban districts (Figure). In fact, the 3 districts (Kangping, Tiexi, Faku) reporting the highest incidence rates were all suburban and industrial districts. The underlying reason was the aggregation of migrant workers in these districts. Shenyang is a hub city in northeastern China where workers from the surrounding rural regions come for job opportunities. These labor workers gather at suburban and industrial districts, and ≈20% of them lack proper vaccination because of limited healthcare access during childhood. Eventually, the aggregation of these susceptible persons caused the adult epidemic in this outbreak.

Although measles incidence in China has decreased sharply since 2010, multiregion epidemics have again been reported, especially among adults, in recent years. The underlying reasons for the Shenyang outbreak in 2014 are limited vaccine coverage and aggregation of susceptible persons. This adult-centered epidemic should serve as a reminder that preventing measles in adults might play an increasing role in future measles elimination efforts.

World Health Assembly and global vaccination partners endorsed the Global Vaccine Action Plan in 2012, and WHO now aims to eliminate measles in 5 of the 6 WHO regions by 2020 (*6*,*7*); the United States first achieved this goal in 2000. However, multiple measles outbreaks were reported in recent years in countries where elimination has been achieved, such as the United States (*8*) and Australia (*9*), mainly because of transmission resulting from international travel and low vaccine coverage in some populations (*10*). China is the most populous country in the world, and eliminating measles in China would help prevent future global transmission events.
